# New pneumothorax complicating successful treatment of persistent air leak by endobronchial valves

**DOI:** 10.1002/rcr2.732

**Published:** 2021-03-07

**Authors:** Yiu Cheong Yeung, Yu Hong Chan, Man Ying Ho, Ming Chiu Chan, Hau Chung Kwok, Wai Cho Yu

**Affiliations:** ^1^ Department of Medicine and Geriatrics Princess Margaret Hospital Hong Kong

**Keywords:** Adverse event, air leak, endobronchial valve, pneumothorax

## Abstract

Endobronchial one‐way valves (EBV) have been proposed as a treatment option for persistent air leak (PAL) complicating spontaneous pneumothorax when surgical intervention is considered not feasible. Published case series showed this form of treatment to be generally safe. We report two such cases in which both achieved immediate cessation of air leak and post‐procedural chest radiograph showed significant collapse of the treated lobe, but developed sudden onset of shortness of breath within 24 h after EBV insertion. Chest radiograph showed continued collapse of the treated lobes with enlarged ipsilateral pneumothorax in one patient and new contralateral pneumothorax in the other. Pulmonologists and thoracic surgeons inserting EBV for treatment of PAL should be aware of this possible and important complication.

## Graphical abstract

The present case report included two cases of pneumothorax after endobronchial valve treatment of persistent air leak (PAL) complicating pneumothorax. The occurrence of this adverse event (the first case showed new ipsilateral pneumothorax and the second case showed new contralateral pneumothorax) shed light to the underlying mechanism of this complication and has not been reported before in the treatment of PAL.

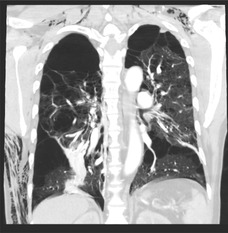

## Introduction

Persistent air leak (PAL) complicating secondary spontaneous pneumothorax is difficult to manage in patients who are not suitable surgical candidates. Non‐surgical options include chemical pleurodesis and various bronchoscopic airway blocking methods. Insertion of endobronchial one‐way valves (EBV) is among the latter and has been shown in published case series to have a fair success rate [1]. Pneumothorax as a complication of EBV insertion has been reported in randomized controlled trials in bronchoscopic lung volume reduction (LVR) for pulmonary emphysema [2, 3]. In contrast, enlarged ipsilateral pneumothorax or new contralateral pneumothorax complicating use of EBV in treating PAL has not been reported.

## Case Report

**Figure 1:**
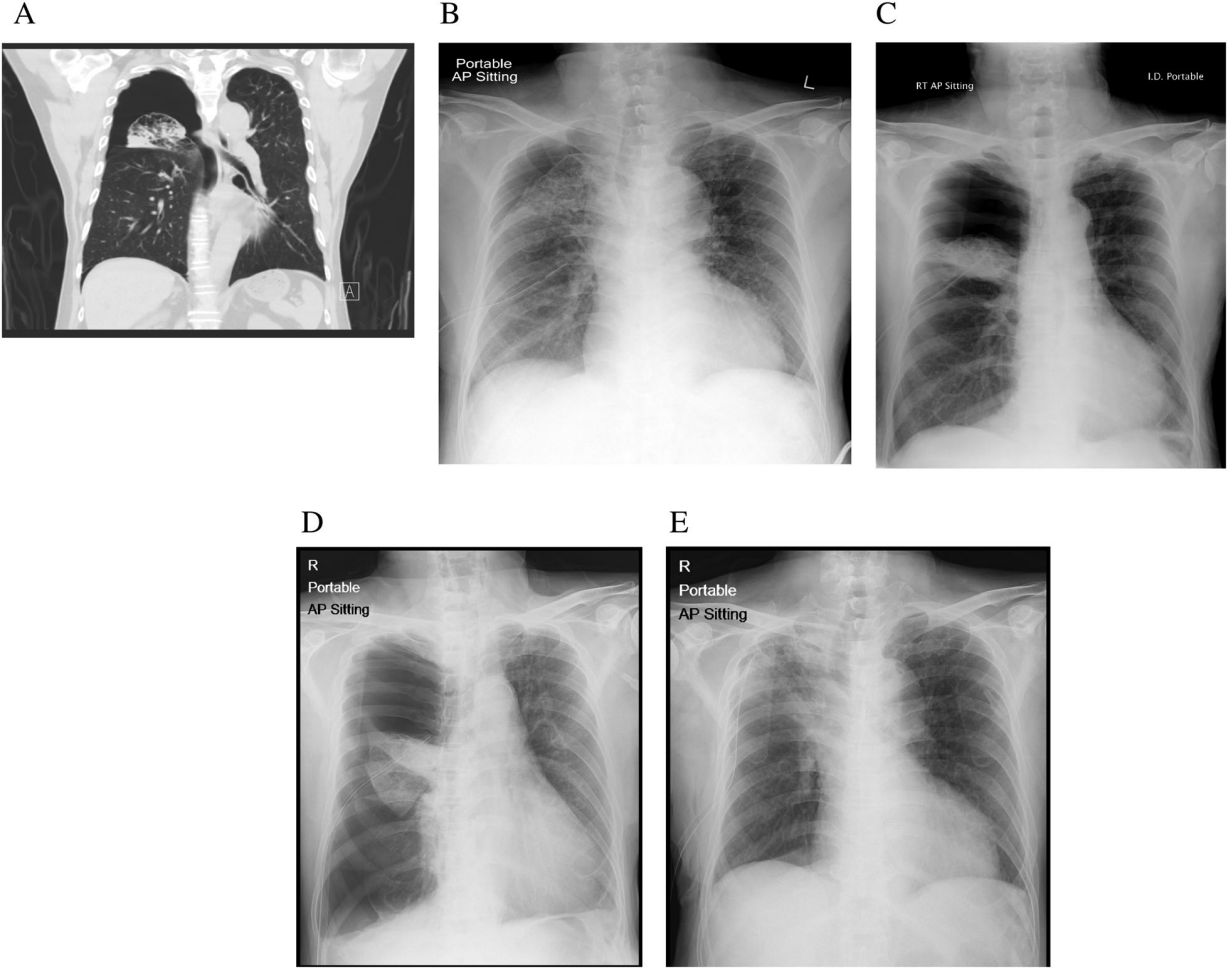
(A) Coronal view of computed tomography (CT) thorax. (B) Chest radiograph (CXR) immediately before endobronchial one‐way valve (EBV) insertion. (C) CXR immediately after EBV insertion. (D) CXR showing enlarged right pneumothorax with old chest tube in situ. (E) CXR after replacement of old chest tube with a new one.

Case 1 was a 70‐year‐old male smoker with a history of chronic obstructive pulmonary disease, atrial flutter, and mitral regurgitation. He presented to the emergency room on 25 October 2019 with a right pneumothorax. A chest drain was inserted with satisfactory lung expansion. Computed tomography (CT) showed right pneumothorax on a background of pulmonary emphysema (Fig. 1A). Air leak persisted and he was not considered a surgical candidate due to poor lung function, as spirometry three years previously showed a forced expiratory volume in 1 sec (FEV_1_) of only 0.92 L (34% predicted) and FEV_1_ to forced vital capacity (FVC) ratio was 0.42. On Day 26, bronchoscopy was performed and balloon occlusion of the right upper lobe (RUL) resulted in immediate cessation of the air leak. Sequential occlusion of individual segmental bronchi of the right upper lobe failed to stop the air leak. Therefore, each of the three segmental bronchi had one EBV inserted, after which air leak stopped immediately. Compared to the pre‐procedural chest radiograph (CXR) (Fig. 1B), the post‐procedural CXR (Fig. 1C) showed near‐complete collapse of the RUL, with pneumothorax still present in the right upper zone, but not in the right lower zone. However, the patient developed sudden increase in shortness of breath 20 h after EBV insertion with clinical feature of tension pneumothorax. CXR showed that the RUL collapse was maintained but there was a large right pneumothorax over both upper and lower zones (Fig. 1D). The old chest drain was replaced by a 20‐F chest drain with improved clinical condition and the CXR showed satisfactory lung expansion (Fig. 1E). There was PAL that was treated with autologous blood patch five days later. Air leak stopped two days afterwards and the chest drain was removed the next day. The EBVs were removed six weeks after its insertion without complication.

**Figure 2:**
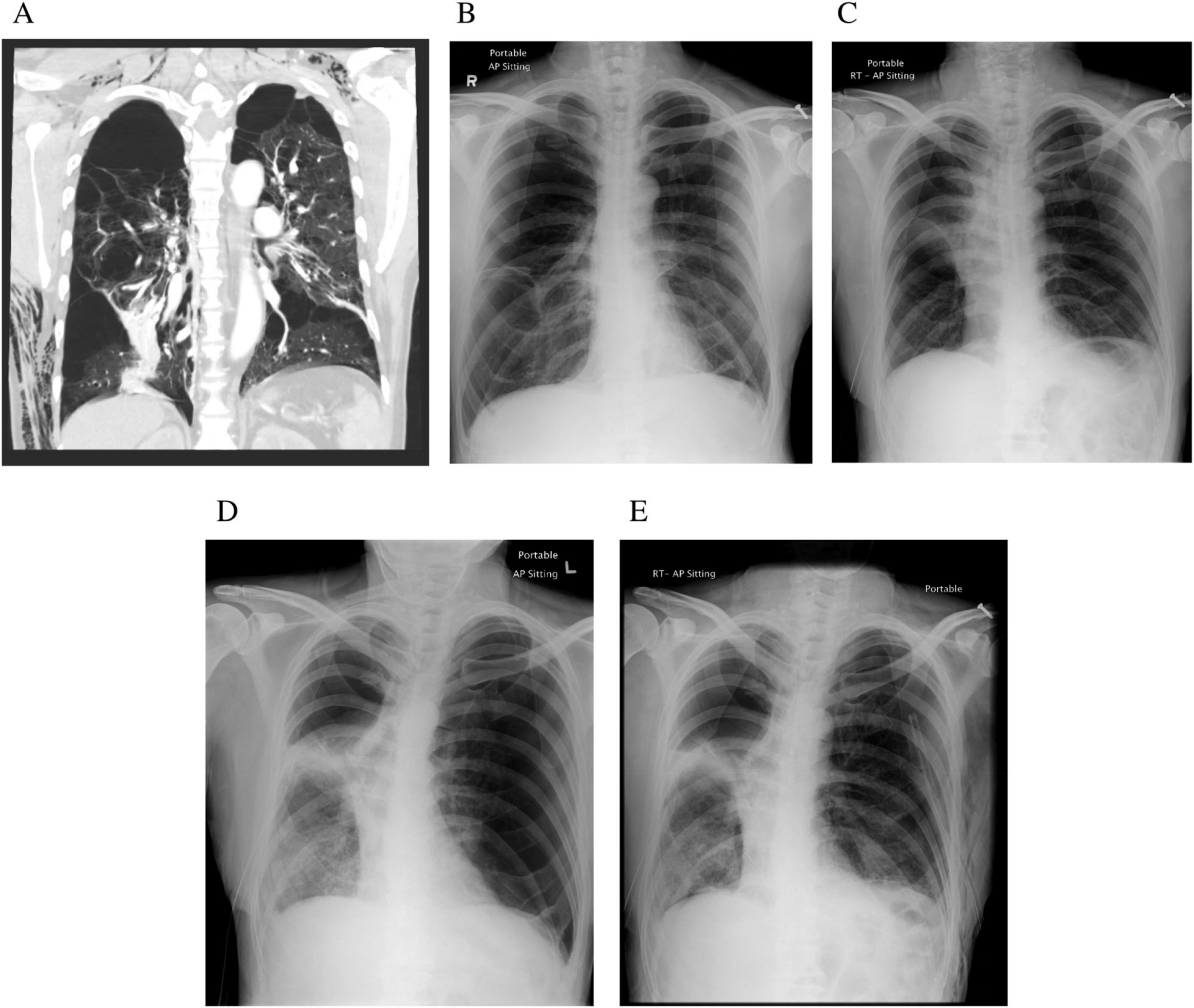
(A) Coronal view of computed tomography (CT) thorax. (B) Chest radiograph (CXR) immediately before endobronchial one‐way valve (EBV) insertion. (C) CXR immediately after EBV insertion. (D) CXR showing new left pneumothorax. (E) CXR showing chest tube inserted for left pneumothorax.

Case 2 was a 56‐year‐old male heavy smoker with no history of prior medical illness. He presented to the emergency room on 11 December 2016 with sudden onset of shortness of breath. CXR showed a right pneumothorax. A chest drain was inserted with satisfactory lung re‐expansion, but with continued air leak. A CT on Day 7 showed diffuse emphysematous changes with large bullae in both lungs (Fig. 2A). He was not considered a surgical candidate in view of the perceived poor lung function as suggested by the CT findings. On Day 25, bronchoscopy was performed with the intention of stopping the air leak by inserting EBVs. Balloon occlusion of the right upper lobe resulted in similar findings to Case 1, so EBVs were inserted into each of the RUL segmental bronchi. Air leak stopped immediately. CXR taken 1 h later (Fig. 2C) when compared to the pre‐procedural CXR (Fig. 2B) showed significant collapse of the right upper lobe. However, the patient developed sudden increase in shortness of breath 12 h after EBV insertion. CXR showed no new findings on the right side but a new pneumothorax on the left side (Fig. 2D), for which a 24‐F chest drain was inserted (Fig. 2E). Subsequently, he suffered from hospital‐acquired pneumonia necessitating removal of the EBVs with recurrence of air leak on the right side. Air leak persisted on both sides. The patient was treated conservatively, and the left PAL stopped spontaneously after three months with the left chest drain removed on 13 April 2017. Air leak on the right side finally stopped following five applications of minocycline chemical pleurodesis, and the right chest drain was removed on 1 June 2017.

## Discussion

We have reported two cases of new pneumothorax occurring within 24 h of EBV deployment for the treatment of PAL complicating secondary spontaneous pneumothorax. In both cases, air leak stopped shortly after deployment of EBVs, associated with radiological collapse of the targeted lobes.

Pneumothorax following EBV insertion for LVR in pulmonary emphysema occurs in 18–29% in experienced centres [2, 3]. It has been reported that when the volume of an emphysematous target lobe is reduced by EBV insertion, the extra volume is redistributed primarily to the ipsilateral untreated lobe(s) [4]. Valipour et al. postulated that such volume changes can cause ruptured pleural blebs or bullae, or parenchymal tears, resulting in a pneumothorax [5]. In the setting of EBV insertion for PAL, success is necessarily accompanied by collapse of the treated lobe and rapid expansion of the untreated lobe(s). The volume of expansion of the untreated lobes may in fact be larger when EBVs are inserted to treat PAL than when used for LVR, due to extrinsic compression of the ipsilateral lobes by air in the pleural space prior to placement. Thus, it is likely that the mechanism for occurrence of pneumothorax proposed by Valipour et al. for bronchoscopic LVR can similarly be applied to EBV treatment for PAL, as in our Case 1.

While the above may provide a possible explanation for a new ipsilateral pneumothorax, it may be more difficult to explain a new contralateral pneumothorax. In our Case 2, we note with interest that collapse of the RUL following EBV insertion was accompanied by shift of the mediastinum towards the right side, suggesting some degree of compensatory expansion of the left lung (Fig. 2B, C). Such observation is also reported by Brown et al., albeit to a much smaller extent than the ipsilateral side [4]. Could this be sufficient to cause lung damage resulting in a pneumothorax? We believe that if the contralateral lung has a similar degree of structural disease, such an event is plausible. Furthermore, onset of a pneumothorax 12 h following EBV deployment appear to be too much of a coincidence. We thus hypothesize that collapse of the RUL following deployment of EBVs resulted in compensatory expansion of the left lung, which resulted in the new left pneumothorax.

To the best of our knowledge, this is the first report of pneumothorax complicating EBV insertion for treating PAL in secondary pneumothorax. Moreover, reports on pneumothorax complicating EBV insertion for LVR have not stated the side of the pneumothorax, and one would assume that most if not all were ipsilateral to the treated lobe. Our report is thus the first to clearly document a contralateral pneumothorax complicating EBV insertion.

In conclusion, pneumothorax can be a complication of EBV insertion for PAL when there is immediate cessation of the air leak. Pulmonologists and thoracic surgeons should discuss the risk of a new air leak when obtaining informed consent. Following a successful procedure, the chest drain should be kept for at least 24 h with close monitoring of the patient.

### Disclosure Statement

Appropriate written informed consent was obtained for publication of this case report and accompanying images.
